# Integrating HIV Prevention and Treatment: From Slogans to Impact

**DOI:** 10.1371/journal.pmed.0020016

**Published:** 2005-01-11

**Authors:** Joshua A Salomon, Daniel R Hogan, John Stover, Karen A Stanecki, Neff Walker, Peter D Ghys, Bernhard Schwartländer

**Affiliations:** **1**Harvard Center for Population and Development Studies, Department of Population and International HealthHarvard School of Public Health, Boston, MassachusettsUnited States of America; **2**The Futures Group International, GlastonburyConnecticutUnited States of America; **3**Joint United Nations Programme on HIV/AIDSGenevaSwitzerland; **4**United Nations Children's Fund, New YorkNew YorkUnited States of America; **5**Global Fund to Fight AIDS, Tuberculosis, and MalariaGenevaSwitzerland; University of Amsterdamthe Netherlands

## Abstract

****Background**:**

Through major efforts to reduce costs and expand access to antiretroviral therapy worldwide, widespread delivery of effective treatment to people living with HIV/AIDS is now conceivable even in severely resource-constrained settings. However, the potential epidemiologic impact of treatment in the context of a broader strategy for HIV/AIDS control has not yet been examined. In this paper, we quantify the opportunities and potential risks of large-scale treatment roll-out.

****Methods and Findings**:**

We used an epidemiologic model of HIV/AIDS, calibrated to sub-Saharan Africa, to investigate a range of possible positive and negative health outcomes under alternative scenarios that reflect varying implementation of prevention and treatment. In baseline projections, reflecting “business as usual,” the numbers of new infections and AIDS deaths are expected to continue rising. In two scenarios representing treatment-centered strategies, with different assumptions about the impact of treatment on transmissibility and behavior, the change in the total number of new infections through 2020 ranges from a 10% increase to a 6% reduction, while the number of AIDS deaths through 2020 declines by 9% to 13%. A prevention-centered strategy provides greater reductions in incidence (36%) and mortality reductions similar to those of the treatment-centered scenarios by 2020, but more modest mortality benefits over the next 5 to 10 years. If treatment enhances prevention in a combined response, the expected benefits are substantial—29 million averted infections (55%) and 10 million averted deaths (27%) through the year 2020. However, if a narrow focus on treatment scale-up leads to reduced effectiveness of prevention efforts, the benefits of a combined response are considerably smaller—9 million averted infections (17%) and 6 million averted deaths (16%). Combining treatment with effective prevention efforts could reduce the resource needs for treatment dramatically in the long term. In the various scenarios the numbers of people being treated in 2020 ranges from 9.2 million in a treatment-only scenario with mixed effects, to 4.2 million in a combined response scenario with positive treatment–prevention synergies.

****Conclusions**:**

These analyses demonstrate the importance of integrating expanded care activities with prevention activities if there are to be long-term reductions in the number of new HIV infections and significant declines in AIDS mortality. Treatment can enable more effective prevention, and prevention makes treatment affordable. Sustained progress in the global fight against HIV/AIDS will be attained only through a comprehensive response.

## Abstract 2

Combining the two approaches of prevention and treatment of HIV/AIDS could avert more than 29 million new HIV infections by 2020

## Introduction

In June 2001, heads of state and government convened a United Nations Special Session on HIV/AIDS and adopted unanimously the “Declaration of Commitment on HIV/AIDS” [1]. In preparation for that session, Schwartländer et al. published an estimate of the resource needs for an expanded global response to the epidemic, which called for around US$10 billion for the fight against HIV/AIDS in 2005 [2]. In 2002, on the occasion of the 14th International AIDS conference in Barcelona, Spain, Stover et al. showed that such an immediate and expanded response in low- and middle-income countries could reverse the course of the HIV/AIDS epidemic and avert nearly 30 million infections through 2010 [3]. Today, more resources are available for the fight against HIV than ever before, but global efforts to confront the epidemic continue to disappoint. Worldwide in 2004, more people were living with HIV and more people died of AIDS than in any previous year [4]. In sub-Saharan Africa, home to two-thirds of all people living with HIV/AIDS, and three out of four people dying from AIDS [4], only one in 50 persons with advanced disease had access to life-saving medicines at the beginning of 2004 [5].

The theme of the 15th International AIDS Conference in Bangkok last summer was timely and relevant. “Access for All” calls for extending to all of those in need both sufficient resources and a set of proven interventions to prevent new infections and save lives through effective treatment. Recent developments in HIV treatment, with simple combination therapies priced at less than US$150 per year—unthinkable just a short time ago—were a major driver of discussions during the conference. Widespread access to effective antiretroviral therapy (ART) for people living with HIV/AIDS is now conceivable even in countries with severely limited resources.

The World Health Organization and its partners in the Joint United Nations Programme on HIV/AIDS have defined an ambitious “3 by 5” target of 3 million people on ART—half of those in most urgent need—by the end of 2005. The potential epidemiologic impact of large-scale roll-out of treatment programs, however, remains uncertain. Experience to date is limited, and comes mostly from Western countries and Brazil. While declines in AIDS mortality in the industrialized world have been impressive [6,7,8], many of these success stories have been accompanied by a resurgence in HIV incidence due to increasing risk behavior as emphasis shifted from prevention to treatment in the 1990s [9,10,11]. Will the extension of ART to millions who suffer from AIDS in developing countries be the long-awaited breakthrough in the response to HIV, or will the emphasis on treatment detract from prevention efforts, and thus hamper AIDS control in the medium and long term? The experience in high-income countries underscores the potential perils of failing to adapt prevention strategies to an environment in which life-saving treatment becomes available on a large scale; however, more favorable outcomes in some settings [12,13] indicate that rising risk behavior is not an inevitable outcome of increased treatment access.

In our previous analysis of the potential benefits of a comprehensive package of preventive interventions [3], we noted that these prevention effects would be achieved only in the presence of wide-scale treatment and political support. The two intervening years have seen a dramatic rise in both momentum and financial resources for ART scale-up, but the potential epidemiologic impact of treatment in the context of a broader strategy for HIV/AIDS control has not yet been examined. In this paper, we quantify the opportunities and potential risks of large-scale treatment roll-out. The results of this analysis will be informative for all regions and countries, independent of the level and stage of the epidemic. However, since three of four deaths from AIDS occur in sub-Saharan Africa, successes and failures in rolling out treatments immediately will have the most dramatic effects in this region. We therefore focus our analyses and discussions in this paper on the HIV epidemics in sub-Saharan Africa**.**

## Methods

### Projections of HIV Epidemics in Sub-Saharan Africa

Baseline projections of HIV epidemics in sub-Saharan Africa have been developed by the Joint United Nations Programme on HIV/AIDS and the World Health Organization based on the most current data available, and in collaboration with epidemiologic experts and analysts within the countries assessed [4]. These “business as usual” forecasts from 2004 to 2020 are characterized by the absence of behavioral change or ART scale-up in future years. Combined with the natural dynamics of the epidemic, these assumptions result in a relatively stable HIV prevalence rate.

To simulate the effects of prevention and treatment on HIV/AIDS incidence, prevalence, and mortality, we first adapted the analytic approach used in the previously described Goals model [3] to allow explicit modeling of treatment effects, and calibrated the model to the baseline projections for three African regions (East, West/Central, and Southern) (see Protocol S1 for more details). In line with the predominant epidemiologic pattern in sub-Saharan Africa of HIV spreading through heterosexual contact, the model divides the sexually active population into five different interacting risk groups: single men, single women, married men, married women, and female sex workers. In sub-Saharan Africa HIV is transmitted via other modes at comparatively low levels, and these modes were therefore not considered in our analyses.

The model includes underlying regional demography, acquisition of HIV and other sexually transmitted infections (STIs), progression from HIV to AIDS, and progression from AIDS to death. Annual risks of HIV infection in each risk group depend on the number of partnerships, the number of sex acts per partnership, HIV prevalence among partners and condom use. These risks are magnified by the presence of other STIs [14] and also vary as a function of the time since infection, with the highest risks during acute infection, followed by lower levels that persist until viral loads rise with the onset of clinical AIDS [15,16,17].

The regional models were calibrated as follows: first, plausible ranges were specified for model parameters governing sexual behavior and biological factors (e.g., transmission risks and cofactor effects of other STIs) based on review of published studies and survey results; second, multiple simulations were undertaken by sampling values from each of the ranges and recalculating the model for each set of sampled parameter values; third, model fit was assessed by comparing modeled prevalence for adult males and females separately to baseline projections through 2020; and fourth, the best-fitting parameter set in each regional model was selected for the purpose of scenario analysis (see Protocol S1).

### Alternative Scenarios for Prevention and Treatment

Potential impacts of prevention efforts at a given coverage level were based on previously published estimates [3] for a comprehensive package of 12 interventions that included mass media campaigns, voluntary counseling and testing, peer counseling for sex workers, school-, youth- and workplace-based programs, condom promotion and distribution, treatment for STIs, and prevention of mother-to-child transmission. The comprehensive package described by Stover and colleagues also included interventions such as harm reduction for injecting drug users and peer outreach for men who have sex with men, which we have not modeled for sub-Saharan Africa. Impacts were captured in terms of changes in condom use, sexual partnerships, treatment-seeking for STIs, and age at first sex. The impacts of treatment included increased survival by a median of 3 y, reductions in transmission probabilities given contact with an infected partner, and behavior change.

We examined a range of alternative scenarios based on various levels and effectiveness of prevention interventions, with and without successful attainment of the 3 by 5 treatment target for sub-Saharan Africa:

#### Baseline (“business as usual”).

Risk behaviors are maintained at current levels, and no treatment scale-up occurs. This is simply the baseline scenario that produces a relatively stable prevalence rate over the duration of the projection, with the number of people living with HIV and the number of new infections rising slowly over time because of population growth.

#### Treatment-centered response.

In two alternative scenarios, the 3 by 5 target of 50% coverage of those in need of treatment by the end of 2005 is attained, and scale-up continues to reach 80% ART coverage of those in need by 2010, maintained at 80% thereafter. In an “optimal ART effects” scenario, we assumed that treatment reduces transmissibility by 99%, and that those under treatment have 50% lower annual partnership numbers and two times higher condom use than other adults. With a response that focuses primarily on treatment, it is assumed that behavior in the general community of infected and uninfected adults is unchanged from the baseline. In an alternative “mixed ART effects” scenario, less optimistic assumptions were made: that treatment reduces transmissibility only to the same levels as in asymptomatic infected individuals (two-thirds reduction from no treatment), and that behavior in treated patients is the same as in other adults. To capture the possibility of behavioral disinhibition in response to treatment availability, we assumed that condom use declines by 10% in both treated patients and the general community, with other behaviors unchanged. The potential for disinhibition is suggested primarily by experience in some developed countries, where condom use increased dramatically in the populations at highest risk prior to the introduction of ART but then declined; the likelihood and magnitude of reductions in condom use in sub-Saharan Africa, where such prevention-induced changes generally are much less prominent today, might be questioned. We therefore considered in sensitivity analyses a variant of this scenario that excludes disinhibition but preserves all other assumptions.

#### Prevention-centered response.

In the absence of wide availability of treatment, reflecting weaker political and social support for HIV control efforts, we modeled a scenario in which the comprehensive prevention package described previously [3] has only partial effectiveness at the population level, and no ART scale-up occurs. As evidence about the magnitude of treatment–prevention interactions remains limited, we considered a reduction of 50% from the full impact as a base case and examined a range of reductions from 25% to 75% in sensitivity analyses.

#### Combined response.

We examined two scenarios combining treatment and prevention efforts, reflecting either optimistic or pessimistic possibilities. In the optimistic scenario, treatment strengthens prevention efforts. ART coverage is the same as in the two treatment-centered scenarios, with optimal assumptions about treatment impact on transmissibility and patient behavior. It is assumed that widespread availability of treatment enables the full impact of prevention efforts to be attained as described by Stover et al. [3]. In a more pessimistic scenario, an emphasis on treatment leads to less effective implementation of prevention. This scenario includes the mixed assumptions about ART effects (excluding disinhibition in the general community), and assumes only 25% attainment of the maximum potential impact of prevention efforts.

Additional scenarios could include pessimistic assumptions about limited ART scale-up levels and timing, emergence of large-scale drug resistance resulting from low adherence, or other possible unintended outcomes of wider treatment. Certainly, large-scale treatment efforts will demand close monitoring of adverse effects. However, experience with treatment programs in developing countries has been encouraging thus far, with reported adherence levels that are at least as high as those in developed countries [18,19].

## Results

**Figure 1:**
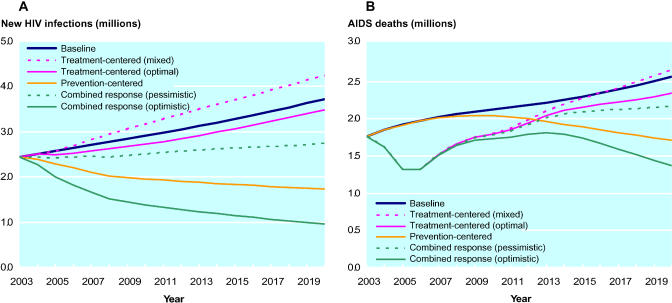
HIV Incidence and AIDS Mortality among Adults in Sub-Saharan Africa, 2003–2020, under Different Intervention Scenarios (A) HIV incidence. (B) AIDS mortality.

In the baseline projections for sub-Saharan Africa, the annual number of new adult HIV infections rises from 2.4 to 3.7 million between 2004 and 2020, and adult AIDS mortality rises from 1.8 to 2.6 million (Figure 1). If scale-up of ART reaches the 3 by 5 target and eventually expands to 80% coverage, without any behavior change in the broader community (treatment-centered response/optimal ART effects), the annual number of new infections could be reduced by up to 6% compared to baseline by 2020. Mortality would initially decline by 33% but long-term trends would converge toward the baseline. We note that total annual death numbers indicate broad trends in mortality but mask more subtle health gains in the form of years added to individuals' lives.

With less optimistic assumptions (treatment-centered response/mixed ART effects), the number of new infections rises, to 4.3 million per year by 2020 (a 14% increase); mortality trends are similar to the optimistic scenario in the short term, but worse in the long term, even compared to the baseline. Excluding the assumption of reduced condom use through disinhibition from the treatment-centered/mixed effects scenario has minimal effect on the results, lowering the number of new infections in 2020 by only 2% compared to the scenario that includes disinhibition.

A prevention-centered response would have greater impact on the number of new infections, lowering annual incidence by more than half by 2020. The long-term mortality trend is more favorable in the prevention-centered scenario than in the treatment-centered scenario because of reduced incidence, but prevention would produce negligible mortality benefits in the near- and mid-term future in comparison to strategies that include ART. Alternative assumptions regarding overall effectiveness in a prevention-centered response produce results that scale as expected, with reductions in annual incidence of 34% to 64% and reductions in annual mortality of 20% to 42% by 2020.

If treatment and effective prevention are scaled up jointly in a combined response, the benefits in terms of both infections and deaths averted could be substantially higher. In an optimistic scenario in which treatment programs support expanded prevention, the annual number of new infections would be 74% lower and annual mortality would be 47% lower by 2020, compared to baseline. It is worth noting that the long-term decline in AIDS deaths is driven more by prevention of new infections than by direct survivorship benefits from ART. In a pessimistic scenario in which a more narrow treatment focus limits effective prevention, the overall benefits are much more modest, with 26% and 16% reductions, respectively, in new infections and mortality by 2020 compared to the baseline.

Prevalence rises by 7% in the optimal and by 27% in the mixed treatment-centered scenarios by 2020, as longer survival for treated patients offsets reductions in new infections through reduced transmissibility (and risk reductions among treated patients in the more optimistic scenario) (Figure S1). In scenarios that include prevention efforts, prevalence declines by 41% in the prevention-centered scenario, by 53% in the optimistic combined response, and by 6% in the pessimistic combined response by 2020.

The total number of infections averted through a combined response would be 29 million over the period 2004 to 2020 if treatment enhances prevention, a benefit that is ten times greater than that of a strategy which focuses on treatment only, even with optimal assumptions, and 51% greater than that of a strategy which focuses on (less effective) prevention alone (Table 1). If a treatment focus limits the effectiveness of prevention, on the other hand, the total number of averted infections between 2004 and 2020 would be 9 million. Similarly, the benefits of a combined response in terms of mortality reductions are considerably higher under optimistic circumstances than the benefits of either treatment only or prevention only, with 10.1 million deaths averted (27%) through 2020 when treatment enhances prevention, compared to 5.0 million (13%) in the optimal-effects treatment only scenario, 3.5 million (9%) in the mixed-effects treatment only scenario, and 4.8 million (13%) with prevention only. Under more pessimistic assumptions about treatment–prevention interactions, the combined response would avert 5.8 million deaths (16%). Table 1 also reports total benefits of the various strategies over the shorter term, in which the ranking of alternatives is similar with regard to the total number of infections averted, but mortality reductions are attributable almost exclusively to treatment.

**Table 1:**
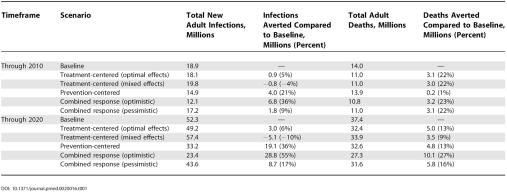
Total New Adult Infections and Deaths in Sub-Saharan Africa, 2004–2010 and 2004–2020, under Different Intervention Scenarios

**Figure 2:**
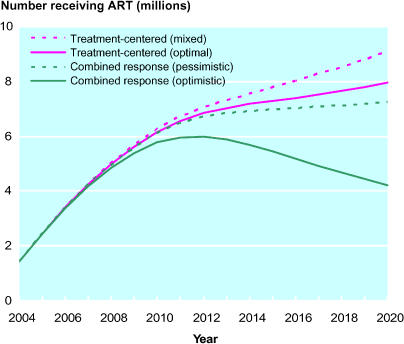
Number of Persons on ART in Sub-Saharan Africa, 2004–2020, under Various Scenarios

Combining treatment with prevention efforts will reduce the resource needs for treatment substantially in the long term (Figure 2). In the various scenarios the numbers of people being treated in 2020 ranges from 9.2 million in the treatment only (mixed effects) scenario, to 4.2 million in the optimistic combined response scenario.

## Discussion

In this paper, we have examined the potential epidemiologic impact of global HIV/AIDS control efforts under a range of alternative scenarios reflecting varying implementation of strategies for prevention and treatment. Although we focus in particular on population health outcomes and epidemiologic trends, we recognize that there are numerous other social, economic, and individual health effects of interventions including ART that are beyond the scope of this analysis. We also restrict our focus in this paper to sub-Saharan Africa, where the overwhelming majority of people living with and dying from HIV/AIDS reside; however, our findings have broader applicability and more general implications in the worldwide fight against HIV/AIDS, which we highlight here.

### The Potential for Treatment to Enhance Prevention Must Be Exploited

Effective prevention requires more than having sufficient funds to offer information and services. It also requires an environment that encourages people to internalize messages about risky behavior and to adopt actual behavior change, and allows people to utilize services such as testing and counseling without fear of stigma or discrimination. Stoneburner and Low-Beer have argued that the supportive social and political environment in Uganda allowed people to discuss AIDS with family members and close friends, which led to greater behavior change than in Kenya or Zambia where most people received information from mass media only [20]. People have little reason to seek HIV testing when a positive result brings only negative consequences, whereas widespread availability of treatment provides a major incentive for people to learn their serostatus.

Involving communities and family members in the delivery of treatment—for example, as treatment monitors—offers unique entry points for effective prevention activities and a lever for population-wide behavior change. Experience with community roll-out of treatment programs has shown, for example, that uptake of voluntary counseling and testing increased by 300% in one year of roll-out in Haiti, and by a factor of 12 in Khayelitsha, South Africa, after treatment introduction [21,22]. A study targeting nine commuter sites in South Africa found the highest levels of condom use, willingness to use a female condom, and willingness to have an HIV test in Khayelitsha, a difference that may be attributed largely to the availability of ART and comprehensive AIDS care [23]. If increased uptake of voluntary counseling and testing is indicative of broader prevention effectiveness where ART is available, we estimate that over 50% more new infections and more than twice as many deaths could be avoided through a combined response compared to prevention alone. In contrast, if a narrow treatment scale-up leads to reduced effectiveness of prevention, short-term mortality reductions will come at the expense of longer-term progress in stemming the tide of the epidemic.

During most of the past 15 years, efforts to address the AIDS epidemic in sub-Saharan Africa have focused on prevention. There have been successes in some countries, but overall these efforts have not achieved their goals. The advent of vastly expanding treatment programs in the coming years, if opportunities to capitalize on broadened political support and community mobilization can be seized, offers the potential to enhance prevention effectiveness and avert many new infections and deaths.

### Only Effective Prevention Will Make Treatment Affordable in the Long Run

While prevention programs are unlikely to achieve full impact in the absence of treatment, so too is the impact of treatment programs reduced if vigorous prevention efforts are absent. Without effective prevention, the number of people requiring care and treatment will grow each year. As more and more people are kept alive with ART the treatment burden will become enormous unless effective prevention reduces the number of people becoming newly infected.

Without effective prevention programs, we project that the number of people receiving treatment will grow to 6.3 million by 2010 and up to 9.2 million by 2020 in Africa alone to achieve 80% coverage of those in urgent need. Meeting this need would require a tremendous increase in financing, human capacity and infrastructure that might not be attainable.

If effective prevention programs are combined with treatment programs, the same level of 80% ART coverage would be achieved by treating 5.8 million in 2010 and 4.2 million in 2020. In other words, the same goal could be attained at a far lower treatment cost and with a much greater chance of sustainability.

### A Successful Global Response Cannot Rely on Either Prevention or Treatment Alone

Over the long term, it is effective prevention that will reduce the burden of illness due to AIDS and the number of people in need of ART. The lessons learned in the industrialized world have to be taken on board. The availability of treatment and the shift in focus away from very effective prevention programs has led to increases in unsafe sexual behavior, STIs, and HIV transmission in some settings [9,10,11]. There is no doubt that effective therapy can extend and improve the quality of life for those who are treated, but it also must be integrated into a comprehensive community response to HIV so that it can enhance the effectiveness of prevention efforts. Long-term, sustained progress in the fight against AIDS demands more than an exclusive focus on either prevention or treatment alone. Prevention makes treatment affordable, and treatment can make prevention more effective.

Countries in sub-Saharan Africa are faced with the most devastating epidemic of our times. We now have the unique opportunity to derive the maximum impact from available resources. The results from our analyses show how potential synergies between prevention and treatment could be translated into considerable health benefits at the population level. But synergies do not mean simply that prevention and treatment are pursued in parallel. When whole communities become involved in the scale-up of treatment access—as will be necessary to achieve the ambitious treatment targets defined by the 3 by 5 campaign—crucial opportunities can be created for increasing their involvement in prevention activities. Only if interactions with patients, family, and community members occasioned by the provision of treatment are also used to reinforce prevention, and only if prevention workers have an opportunity to refer those in need to care and treatment, will we move at last from slogans to impact.

## Supporting Information

Figure S1 Adults Living with HIV/AIDS in Sub-Saharan Africa, 2003–2020, under Different Intervention Scenarios (80 KB EPS).

Protocol S1 Technical Appendix (172 KB PDF).

**Patient Summary**

**Background**

Infections from HIV continue to increase, especially in sub-Saharan Africa. The World Health Organization has a plan to get more than 3 million people on treatment by 2005 (the “3 by 5” initiative); however, the overall effect of this plan on the population's health is uncertain, and will depend on the balance between treatment and prevention efforts.

**What Did the Researchers Do?**

They tried to predict the number of new infections and deaths each year in sub-Saharan Africa from now until 2020 depending on whether control efforts focused on prevention, treatment, or both. What they found was that by far the most effective way of decreasing new infections and deaths was to combine the two approaches, and that by doing so more than 29 million new infections and 10 million deaths might be prevented compared with continuing at current levels of prevention and care.

**Why Is This Information Important, and Who Will Use It?**

Despite the huge amounts of money directed at HIV/AIDS, because the problem is so vast, the resources are not enough. Hence it is important to target these resources effectively. Policy makers around the world could use information like this to decide where best to direct attention and funding to combat HIV/AIDS.

**Where Can I Find More Information?**

Joint United Nations Programme on HIV/AIDS, AIDS epidemic update, December 2004: http://www.unaids.org/wad2004/report.html

World Heath Organization, 3 by 5 Initiative: http://www.who.int/3by5/

Global HIV Prevention Working Group: http://www.kff.org/hivaids/hivghpwgpackage.cfm

## Abbreviations

ART: antiretroviral therapy
STI: sexually transmitted infection
