# Supplementary material for: Integrating HIV Prevention and Treatment: From Slogans to Impact
Source: PLoS Med. 2005 Jan 11;2(1):e16. doi: 10.1371/journal.pmed.0020016 (PMC543462; doi:10.1371/journal.pmed.0020016)
Supplement: Protocol S1 — (172 KB PDF). [file pmed.0020016.sd001.pdf]

Integrating HIV prevention and treatment: from slogans to impact

Joshua A Salomon, Daniel R Hogan, John Stover, Karen A Stanecki, Neff Walker, Peter D Ghys, Bernhard Schwartländer

Protocol S1. Technical Appendix

We developed a mathematical model of HIV/AIDS, adapted from the Goals model described previously [1,2]. The model includes underlying demography, sexual mixing between defined risk groups, transmission of HIV infections, progression from HIV to AIDS and AIDS to death, and transmission of other sexually transmitted infections (STI). A range of different interventions may be incorporated, with impacts on risk behaviors and progression rates. The model is implemented in an Excel spreadsheet. The @RISK package [3] is used to undertake multiple simulations of the model through sampling of uncertain parameter ranges, which allows both calibration of parameter values by fitting to observed data and uncertainty analysis of model outputs.

Model structure

Demographic and behavioral model

In this study, the model has been calibrated to 3 regions in sub-Saharan Africa (East, West/Central and Southern) as described below. The model focuses on adult populations (ages 15 to 49 years), a subset of whom are sexually active. Population size in each year is based on country-specific projections from the 2002 revision of the United Nations population estimates and projections [4], to which the impact of AIDS is added using the Spectrum package [5], and then aggregated to regional level. New entrants into the adult population each year are assumed to be uninfected. Baseline population projections are modified in scenarios that include treatment to account for increased survivorship of treated AIDS patients where relevant.

The model divides the sexually active population into five interacting risk groups: single men, single women, married men, married women, and female sex workers (FSW). Four types of partnerships may be formed: between single men and single women, between married men and married women, and between single or married men and FSW. In line with the predominant epidemiologic pattern in sub-Saharan Africa of HIV spread through heterosexual contact, we exclude men who have sex with men from the analysis. Single men and married men have distinct probabilities of visiting sex workers in addition to their partnerships with single or married women. Group sizes, numbers of partnerships, and number of sexual acts per partnership determine the total number of acts in each risk-group pairing.

To balance the total number of acts between males and females in the various types of partnerships, the distribution of women across risk groups is not entered as an input into the model, but rather calculated based on other inputs. Male demand for sex work determines the number of FSW in the population up to a specified limit:

total demand for sex work = (number of active single men × proportion who visit sex workers × number of visits per year) + (number of active married men × proportion who visit sex workers × number of visits per year)

number of FSW = total demand for sex work / average annual number of visits per sex worker

 © 2005 Salomon et al.
DOI: 10.1371/journal.pmed.0020016.sd001

When the total demand would produce a larger number of FSW than the specified population limit, male demand is scaled down, preserving the ratio between demand from single and married men. The number of married women is determined based on the number of married men:

number of married women = number of married men × average number of wives per husband

The number of single women is then calculated as the residual:

single women = total women – married women – FSW

The number of acts per partnership for single men is determined by demand from single women. Within risk groups, condom use and sex acts per year may vary between those with or without clinical AIDS, and by treatment status among those with AIDS.

Disease models

The HIV disease model distinguishes five states: uninfected, primary HIV infection, post-primary / pre-AIDS infection, untreated AIDS, and treated AIDS. For the purpose of this analysis, the label *AIDS* is intended as shorthand for advanced disease rather than as a strict clinical definition. In the model, this characterization distinguishes those persons regarded as being in most urgent need of treatment according to treatment initiation guidelines from WHO.

Progression from HIV to AIDS is calculated using a Weibull function with parameters consistent with recommendations from the UNAIDS Reference Group on Estimates, Modelling and Projections (see below) [6].

The STI disease model includes three states: uninfected, genital ulcerative disease (GUD), and non-ulcerative disease (non-GUD). For purposes of parameterizing initial prevalence, transmissibility, and duration of STI states, GUD is assumed to include syphilis, chancroid, and herpes simplex virus-2; non-GUD includes chlamydia, gonorrhea, and trichomoniasis. For the initial year of 1999, HIV prevalence is based on UNAIDS/WHO estimates, and STI prevalence is based on the Global Burden of Disease Study [7].

Model transitions

HIV transmission

For each of the five risk groups, the probability of infection during each one-year period is calculated, and this probability is multiplied by the uninfected population at the start of the year to calculate the annual number of new infections. A binomial model of HIV transmission is used, based on a modification of the equation originally presented by Weinstein et al. [8] and implemented in the AVERT model [9] and Goals [2], as follows:

$$P_{is} = 1 - \prod_k \prod_j \left[1 - b_{isj}\left(1 - c_k\right)\right]^{a_k N_k x_{jk}}$$

where i indicates risk group, s indicates STI state (uninfected, GUD, non-GUD), k indicates partner type, j indicates HIV state (uninfected, primary HIV infection, post-primary / pre-AIDS infection, untreated AIDS, and treated AIDS), and

P_{is} = one-year probability of infection for an individual of risk group i and in STI state s

b_{isj} = probability of transmission for an individual in risk group i and in STI state s per (unprotected) contact with a person in HIV state j.

c_k = effective condom use during contacts with partner type k

a_k = average number of sexual contacts per year per partnership of type k

N_k = number of partners per year of type k

x_{jk} = proportion of contacts with k-type partners in which partners are in HIV state j

Married and single women have only one partner type, so the equation may be simplified for these groups to:

$$P_{is} = 1 - \prod_j \left[1 - b_{isj}\left(1 - c_k\right)\right]^{a_k N_k x_{jk}}$$

The proportions of contacts in different HIV states are determined by the prevalence of HIV infection and AIDS in the partner group at the beginning of the year, and the level of treatment coverage. Note that in these equations we include "uninfected" as an HIV state, but the term in the product that includes this partner type is simply 1 since the associated transmission probability is 0.

Our modified binomial specification provides a close approximation of the risks determined by the original formulation. The adapted formula has the advantage of allowing explicit modeling of behavior change associated with partners in a particular HIV state; for example, the behavior of treated individuals may be targeted by interventions separately from behavior among untreated individuals, and reduced transmissibility through treatment is linked specifically to sexual contacts with treated partners.

Per-contact transmission probabilities (b) vary according to the HIV disease stage of the partner (modeled in "bathtub" fashion with highest rates during primary infection, low rates during post-primary HIV, and medium-high rates during clinical AIDS) (see Table S2) [10]. Probability of transmission also depends on the STI state of the uninfected partner (modeled as cofactor effects on susceptibility to infection from having GUD or non-GUD). Note that although b is indexed by risk group i, the transmission probabilities are constant for the various risk groups of a given sex, conditional on HIV disease state and STI state. HIV prevalence within each risk group is adjusted each year to reflect transitions from one risk group to another (e.g. movements in and out of commercial sex work, or changes in marital status).

STI transmission

Prevalence of GUD and non-GUD is computed in monthly cycles due to shorter durations of infection, based on net changes through new infection and remission. For a given category of STI, prevalence in risk group i (S_i) is computed in monthly time steps (t) as follows:

$$S_i(t+1) = S_i(t)e^{r_i}$$

The rate of change in prevalence of an STI (by category) in risk group i is given by:

$$r_i = \left[1 - \prod_k \left(1 - g_i\left(1 - c_k\right)\right)^{a_k N_k Y_k}\right]\left(1 - S_i(t)\right) - \left(\frac{1}{d}\right) S_i(t)$$

where

g_i = probability of transmission for an individual in risk group i per (unprotected) contact with a person having an STI (category-specific)

c_k = effective condom use during contacts with partner type k

a_k = average number of sexual contacts per year per partnership of type k

N_k = number of partners per year of type k

Y_k = prevalence of STI (category-specific) among partner type k

d = average duration of an STI (category-specific), in months

The average duration of an STI is computed based on input parameters defining the duration of treated infections, the duration of untreated infections, and the proportion of infections that are treated, which may vary in different prevention scenarios. The reciprocal of the duration approximates the exit rate from the class of prevalent infections over a one-month time period.

Progression to AIDS and death

Newly infected individuals are exposed to survivorship curves that govern progression from HIV to AIDS and from AIDS to death. Consistent with recommendations from the UNAIDS Epidemiology Reference Group [6], HIV-to-AIDS progression is based on a Weibull function with median progression time to AIDS of 7.5 and 8.5 years for men and women, respectively; median survivorship with AIDS is 1 year in the absence of treatment; and antiretroviral therapy confers a median of 3 years of additional survivorship.

The prevalent cohort of HIV-infected individuals in the first year of the model (1999) is subject to a different survivorship curve since this cohort includes surviving members of multiple incident cohorts from a range of prior years, each with different conditional survival probabilities in any given calendar year. The initial survivorship schedule was computed by applying the standard survivorship function described above to UNAIDS/WHO estimates of incidence in each year since the start of the epidemic and then summing the remaining survivors from all previous cohorts in each of the years following 1999.

Model calibration

Overview

Baseline projections of country-specific HIV epidemics in sub-Saharan Africa were developed by UNAIDS and WHO and aggregated into the three regions used in this analysis. We specified ranges around uncertain behavioral and biological parameters in the present model based on published studies and survey data. Values were sampled randomly from these ranges in order to undertake multiple model simulations, and modeled outcomes from each sampled parameter set were assessed in terms of fit to the baseline projections of male and female prevalence over the period 1999 to 2020.

Baseline projections

Baseline projections are taken from UNAIDS/WHO country estimates. Details of the methods used to develop these estimates are published elsewhere and summarized here [5,11,12]. HIV prevalence among pregnant women attending antenatal clinics was used to estimate prevalence in all adults between ages 15

and 49 years [12]. Epidemic curves were fit to prevalence data from pregnant women for past years, separately for urban areas and rural areas, using the Estimation and Projection Package (EPP) [11], with adjustments for representativeness of surveillance sites in rural areas. National epidemic curves were estimated by applying the urban/rural population distribution to the separate urban and rural epidemic curves. Baseline projections to 2020 in EPP were computed under the assumption of no future behavioral change. The Spectrum software package [5] was used to derive estimates of adult incidence and mortality based on the EPP prevalence estimates.

Parameter ranges

Ranges around behavioral parameters in the model were specified based on review of the literature and data from the Demographic and Health Surveys. Ranges were developed for East and Southern Africa combined, and West/Central separately, although there is considerable overlap in the two sets for most parameters given persistent uncertainties and limited data (Table S1). For biological parameters, the same ranges were used as starting points for simulations in all regions (Table S2).

AIDS-specific parameters

Persons with clinical AIDS were assumed to have half as many partners per year as the rest of the sexually active population, as studies have reported that AIDS-defining illnesses lead to increased morbidity and reduced sexual behavior [13,14]. The transmissibility of untreated AIDS patients was set at 3 times that of persons with post-primary / pre-AIDS infection, based on the study by Quinn et al. showing that transmission probabilities per sex act increased by a factor of 2.45 for each log increment in viral load [15], and the increase in viral load by a factor of 10 or more between asymptomatic HIV and AIDS observed in numerous studies [16,17,18].

Simulations and goodness-of-fit

For each regional model, we used @RISK software to undertake 10,000 simulations. In each simulation, parameter values were sampled randomly from uniform distributions defined by the ranges described above. Projected prevalence numbers in the simulations, from 1999 to 2020 and by gender, were compared to the corresponding baseline projections, and goodness-of-fit was computed as the squared percent deviation of modeled prevalence from baseline. The parameter set that minimized the maximum deviation across all years was identified as the best-fit set of parameter values and used for scenario analysis. Because the calibration identified the parameters that provided the best overall prevalence fit for a 22-year period, the selected values did not produce an exact match to reference estimates of incidence and mortality in the year 2003 (deviations were approximately 7% and 10% for incidence and mortality, respectively). For comparability, we have therefore normalized incidence and mortality figures to match the 2003 reference estimates of incidence and mortality.

Intervention effects

For prevention interventions, impact was computed as described previously by Stover et al. [2], starting from literature-based estimates of effects of individual interventions on specific behaviors (Table S3). Derivation of the estimates of intervention effects is detailed in Bollinger et al. [19].

Individuals with advanced disease (labeled as *AIDS*) may be treated in the model, consistent with the primary focus of delivering ART to those in most urgent need. Treated patients are allowed different sexual behavior and transmissibility than untreated patients. Indirect effects of treatment on sexual behavior of untreated patients due to changes in supply and demand dynamics are implemented as adjustments to the distribution of sexual contacts for those without AIDS as follows: the number of

contacts with non-AIDS partners is the same as in the counterfactual of no treatment; the number of contacts with partners having untreated AIDS is reduced in proportion to the reduction in the population of untreated AIDS patients (due to delivery of treatment); in instances where excess demand is generated by treatment of AIDS patients (through prolonged survivorship or behavior change), the excess demand is satisfied by pre-AIDS partners in proportion to their relative contributions to overall demand.

Scenarios

Baseline (business as usual).

Risk behaviors are maintained at current levels (i.e., no increase in prevention efforts), and no treatment scale-up occurs. This is simply the baseline scenario that produces a relatively stable prevalence rate over the duration of the projection, with the number of people living with HIV and the number of new infections rising slowly over time because of population growth.

Treatment-centered response (optimal ART effects).

In this paper, we have modeled scenarios in which the targets of 3 by 5 are obtained: 50% treatment coverage in sub-Saharan Africa implies around 2 million treated patients in this region by 2005. We also adopt the optimistic assumption that 80% coverage is attained by 2010, as our objective is to examine the potential epidemiologic impact of widespread delivery of ART. This scenario includes an optimistic set of assumptions for the direct effect of ART on reducing incidence: very low transmissibility and low risk behavior among treated individuals.

We assume that treatment reduces transmissibility by 99%, which is consistent with reductions in viral load observed in treatment trials, combined with the relationship between transmissibility and viral load observed by Quinn et al. [15]. In the Quinn study, no transmission occurred below 1500 copies/ml; in other modeling work, Gray et al. [20] assume a 96% reduction in transmissibility associated with a change in viral load from >38,500 to <1,700 copies/ml. Viral load reductions of this magnitude have been reported in African settings; for example, Landman et al. [21] reported 95% of patients in Dakar on HAART below 500 copies/ml after 6 months, and Coetzee et al. [22] found 70% of patients in Khayelitsha on HAART below 400 copies/ml after two years.

For the optimal-effects scenario, we also assume that treated patients will have half as many partnerships as those without AIDS (i.e. maintain the same level of reduced sexual behavior as untreated clinical AIDS patients) and will double their condom use due to education and counseling during treatment, consistent with findings in a recent study in Cote d’Ivoire [13].

Treatment-centered response (mixed ART effects).

ART coverage assumptions are as in the previous scenario, but less optimistic assumptions are that treatment reduces transmissibility only to the same levels as in asymptomatic infected individuals (two-thirds reduction from no treatment), and that treated patients resume partnership rates equal to other adults. To capture the possibility of behavioral disinhibition in response to treatment availability, it is assumed that condom use declines by 10% in both treated patients and the general community, a substantially smaller reduction than that observed among men who have sex with men in San Francisco, where the proportion reporting unprotected sex doubled from 1994 to 1999 [23].

Prevention-centered response.

In the absence of wide availability of treatment, reflecting weaker political and social support for HIV control efforts, we consider a scenario in which the comprehensive prevention package described previously [2] has only partial (50%) effectiveness at the population-level, and no ART scale-up occurs. We conducted an analysis of the studies used to estimate the impact of interventions on behavior change. The effectiveness of prevention interventions in promoting behavior change shows a wide range of effects with some interventions showing no effect and others showing effects over twice as large as the average for all interventions of that type. While there are many reasons that interventions will have different effects, some of this difference is likely due to varying levels of political support, stigma and access to treatment. We examine alternative assumptions about the level of attainment of prevention effectiveness (ranging from 25% to 75% of maximum impact) in sensitivity analyses. Maximum impact is computed under the following assumptions about target coverage rates: 100% for mass media, voluntary counseling and testing and school-based programs; 75% for STI treatment; 60% for FSW peer counseling, condom social marketing and public sector condom distribution; and 50% for workplace programs and prevention of mother-to-child transmission.

Combined response (optimistic).

ART coverage is the same as in the 2 treatment-centered scenarios, with the *optimal ART effects* assumptions about treatment impact on transmissibility and patient behavior, and it is assumed that widespread availability of treatment enables the full impact of prevention efforts to be attained as described by Stover et al. [2].

Combined response (pessimistic).

ART coverage is the same as in the 2 treatment-centered scenarios, with the *mixed ART effects* assumptions about treatment impact on transmissibility and patient behavior, excluding disinhibition. In this scenario, increased emphasis on treatment leads to less effective implementation of prevention efforts, which we model by assuming that the prevention package will attain only 25% of its maximum impact.

References

1. Stover J, Bollinger L, Cooper-Arnold K (2003) Goals model: for estimating the effects of resource allocation decisions on the achievement of the goals of the HIV/AIDS strategic plan. Glastonbury: The Futures Group International. Available: http://www.futuresgroup.com. Accessed 5 December 2004.
2. Stover J, Walker N, Garnett GP, Salomon JA, Stanecki KA, et al. (2002) Can we reverse the HIV/AIDS pandemic with an expanded response? Lancet 360(9326):73-77.
3. Palisade Corporation (2004) @RISK 4.5 Professional. New Field, NY: Palisade Corporation.
4. Population Division of the Department of Economic and Social Affairs of the United Nations Secretariat (2003) World Population Prospects: The 2002 Revision. New York: United Nations.
5. Stover J (2004) Projecting the demographic consequences of adult HIV prevalence trends: the Spectrum Projection Package. Sex Transm Infect 80(Suppl 1): i14-i18.
6. UNAIDS Reference Group on Estimates, Modelling and Projections (2002) Improved methods and assumptions for estimation of the HIV/AIDS epidemic and its impact: recommendations of the UNAIDS Reference Group on Estimates, Modelling and Projections. AIDS 16(9): W1-16.
7. World Health Organization. WHO Statistical Information System (WHOSIS): Global Burden of Disease Project. Available: http://www.who.int/evidence/bod. Accessed 5 December 2004.
8. Weinstein MC, Graham JD, Siegel JE, Fineberg HV (1989) Cost-effectiveness analysis of AIDS prevention programs: concepts, complications, and illustrations. In: Turner CF, Miller HG, Moses LE, eds. Confronting AIDS: sexual behavior and intravenous drug use. Washington: National Academy Press. pp. 471-499.
9. Rehle TM, Saidel TJ, Hassig SE, Bouey PD, Gaillard EM, Sokal DC (1998) AVERT: a user-friendly model to estimate the impact of HIV/sexually transmitted disease prevention interventions on HIV transmission. AIDS 12(Suppl 2):S27-S35.
10. Koopman JS, Jacquez JA, Welch GW, Simon CP, Foxman B, et al. (1997) The role of early HIV infection in the spread of HIV through populations. J Acquir Immune Defic Syndr Hum Retrovirol 14(3):249-258.
11. Ghys PD, Brown T, Grassly NC, Garnett G, Stanecki KA, et al. (2004) The UNAIDS Estimation and Projection Package: a software package to estimate and project national HIV epidemics. Sex Transm Infect 80(Suppl 1): i5-i9.
12. Grassly NC, Morgan M, Walker N, Garnett G, Stanecki KA, et al. (2004) Uncertainty in estimates of HIV/AIDS: the estimation and application of plausibility bounds. Sex Transm Infect 80(Suppl 1): i31-i38.
13. Moatti JP, Prudhomme J, Traore DC, Juillet-Amari A, Akribi HA, et al. (2003) Access to antiretroviral treatment and sexual behaviours of HIV-infected patients aware of their serostatus in Cote d'Ivoire. AIDS 17(Suppl 3):S69-S77.
14. Deschamps MM, Pape JW, Hafner A, Johnson WD, Jr. (1996) Heterosexual transmission of HIV in Haiti. Ann Intern Med 125(4):324-330.
15. Quinn TC, Wawer MJ, Sewankambo N, Serwadda D, Li C, et al. (2000) Viral load and heterosexual transmission of human immunodeficiency virus type 1. Rakai Project Study Group. N Engl J Med 342(13):921-929.

16. Lyles CM, Dorrucci M, Vlahov D, Pezzotti P, Angarano G, et al. (1999) Longitudinal human immunodeficiency virus type 1 load in the italian seroconversion study: correlates and temporal trends of virus load. J Infect Dis 180(4):1018-1024.

17. Hubert JB, Burgard M, Dussaix E, Tamalet C, Deveau C, et al. (2000) Natural history of serum HIV-1 RNA levels in 330 patients with a known date of infection. The SEROCO Study Group. AIDS 14(2):123-131.

18. Sabin CA, Devereux H, Phillips AN, Hill A, Janossy G, et al. (2000) Course of viral load throughout HIV-1 infection. J Acquir Immune Defic Syndr 23(2):172-177.

19. Bollinger L, Cooper-Arnold K, Stover J (2004) Where are the gaps? The effects of HIV-prevention interventions on behavioral change. Stud Fam Plann 35(1):27-38.

20. Gray RH, Li X, Wawer MJ, Gange SJ, Serwadda D, et al. (2003) Stochastic simulation of the impact of antiretroviral therapy and HIV vaccines on HIV transmission; Rakai, Uganda. AIDS 17(13):1941-1951.

21. Landman R, Schiemann R, Thiam S, Vray M, Canestri A, et al. (2003) Once-a-day highly active antiretroviral therapy in treatment-naive HIV-1-infected adults in Senegal. AIDS 17(7):1017-1022.

22. Coetzee D, Hildebrand K, Boulle A, Maartens G, Louis F, et al. (2004) Outcomes after two years of providing antiretroviral treatment in Khayelitsha, South Africa. AIDS 18(6):887-895.

23. Katz MH, Schwarcz SK, Kellogg TA, Klausner JD, Dilley JW, et al. (2002) Impact of highly active antiretroviral treatment on HIV seroincidence among men who have sex with men: San Francisco. Am J Public Health 92(3):388-394.

24. United States Agency for International Development & Macro International Inc. Demographic and Health Surveys. Available: http://www.measuredhs.com. Accessed 5 December 2004.

25. Asamoah-Adu A, Weir S, Pappoe M, Kanlisi N, Neequaye A, et al. (1994) Evaluation of a targeted AIDS prevention intervention to increase condom use among prostitutes in Ghana. AIDS 8(2):239-246.

26. Morison L, Weiss HA, Buve A, Carael M, Abega SC, et al. (2001) Commercial sex and the spread of HIV in four cities in sub-Saharan Africa. AIDS 15(Suppl 4):S61-S69.

27. Connolly CA, Ramjee G, Sturm AW, Abdool Karim SS (2002) Incidence of Sexually Transmitted Infections among HIV-positive sex workers in KwaZulu-Natal, South Africa. Sex Transm Dis 29(11):721-724.

28. Ghys PD, Diallo MO, Ettiegne-Traore V, Kale K, Tawil O, et al. (2002) Increase in condom use and decline in HIV and sexually transmitted diseases among female sex workers in Abidjan, Cote d'Ivoire, 1991-1998. AIDS 16(2):251-258.

29. Kaul R, Kimani J, Nagelkerke NJ, Fonck K, Keli F, et al. (2002) Reduced HIV risk-taking and low HIV incidence after enrollment and risk-reduction counseling in a sexually transmitted disease prevention trial in Nairobi, Kenya. J Acquir Immune Defic Syndr 30(1):69-72.

30. Carael M (1995) Sexual behavior. In: Cleland J, Ferry B, eds. Sexual behaviour and AIDS in the developing world. New York: Taylor & Francis. pp. 75-123.

31. Wilson D, Chiroro P, Lavelle S, Mutero C (1989) Sex worker, client sex behaviour and condom use in Harare, Zimbabwe. AIDS Care 1(3):269-280.

32. Voeten HA, Egesah OB, Ondiege MY, Varkevisser CM, Habbema JD (2002) Clients of female sex workers in Nyanza province, Kenya: a core group in STD/HIV transmission. Sex Transm Dis 29(8):444-452.

33. Quinn TC, Wawer MJ, Sewankambo N, Serwadda D, Li C, et al. (2000) Viral load and heterosexual transmission of human immunodeficiency virus type 1. Rakai Project Study Group. N Engl J Med 342(13):921-929.

34. Royce RA, Sena A, Cates W, Jr., Cohen MS (1997) Sexual transmission of HIV. N Engl J Med 336(15):1072-1078.

35. Korenromp EL, Bakker R, Gray R, Wawer MJ, Serwadda D, et al. (2002) The effect of HIV, behavioural change, and STD syndromic management on STD epidemiology in sub-Saharan Africa: simulations of Uganda. Sex Transm Infect 78(Suppl 1):i55-i63.

36. Venkataramana CB, Sarada PV (2001) Extent and speed of spread of HIV infection in India through the commercial sex networks: a perspective. Trop Med Int Health 6(12):1040-1061.

37. Gray RH, Wawer MJ, Brookmeyer R, Sewankambo NK, Serwadda D, et al. (2001) Probability of HIV-1 transmission per coital act in monogamous, heterosexual, HIV-1-discordant couples in Rakai, Uganda. Lancet 357(9263):1149-1153.

38. Fleming DT, Wasserheit JN (1999) From epidemiological synergy to public health policy and practice: the contribution of other sexually transmitted diseases to sexual transmission of HIV infection. Sex Transm Infect 75(1):3-17.

39. Robinson NJ, Mulder DW, Auvert B, Hayes RJ (1997) Proportion of HIV infections attributable to other sexually transmitted diseases in a rural Ugandan population: simulation model estimates. Int J Epidemiol 26(1):180-189.

40. Anderson RM (1999) Transmission dynamics of sexually transmitted infections. In: Holmes KK, Sparling PF, Mardh P, Lemon SM, Stamm WE, et al., eds. Sexually transmitted diseases, 3d edition. New York: McGraw-Hill. pp. 25-37.

41. Kasper DL, Braunwald E, Fauci AS, Hauser SL, Longo DL, et al., eds. (2004) Harrison's Principles of internal medicine, 16th edition. New York: McGraw-Hill.

Table S1. Ranges for behavioral parameters in the model.

Parameter	East and South Min	East and South Max	West/Central Min	West/Central Max	References
Sexually active population					
Male (proportion of all adult males)	0.67	0.84	0.63	0.87	[24]
Female (relative proportion)[a]	0.88	0.93	0.87	0.91	[24]
Proportion married among sexually active males	0.60	0.75	0.55	0.83	[24]
Partners per year					
Married males (excluding FSW partners)	1.07	1.17	1.1	1.34	[24]
FSW	500	1200	300	1200	[25-29]
Single females	2	3	2	3	[24]
Married females	1	1	1	1	assumption
Probability man visits FSW					
Single	0.03	0.13	0.04	0.12	[26,30]
Married	½ single		½ single		[24]
Number of visits to FSW per year					
Single male	25	89	25	89	[31,32]
Married male	25	89	25	89	[31,32]
Acts per partnership					
Single male	7	17	7	17	[24]
Married female	30	70	20	60	[24,30]
Risk group transitions (annual probability)					
Single male – married male	0	0.2	0	0.2	assumption
FSW – single female	0	0.2	0	0.2	[26-29]
Single female – married female	0	0.2	0	0.2	assumption
FSW maximum proportion of female population[b]	0.01	0.014	0.019	0.02	[26]

Abbreviation: FSW = female sex worker

[a] Ratio of active proportion of all adult females to active proportion of all adult males.

[b] Male demand determines the number of sex workers until FSW proportion reaches this level, after which male demand is scaled downwards, preserving the ratio between demand from single v. married men.

Table S2. Ranges for biological parameters in the model.

Parameter	Min	Max	References
HIV transmission probability (per act)			
Male – female[a]	1x	3x	[33-36]
Female – male	0.0008	0.0015	[37]
Primary infection cofactor[b]	10	30	[10,35]
GUD cofactor			
Male – female	2	15	[35,38]
Female – male	2	15	[35,38]
Non-GUD cofactor			
Male – female	2	5	[35,38]
Female – male	2	5	[35,38]
GUD transmission probability (per act)			
Male – female	0.20	0.30	[35,39]
Female – male	0.10	0.20	[35,39]
Non-GUD transmission probability (per act)			
Male – female	0.15	0.25	[35,39]
Female – male	0.10	0.20	[35,39]
Duration (years)			
GUD, untreated	0.04	0.22	[35,39,40]
Non-GUD, untreated	0.15	0.30	[35,39,40]
GUD, treated	0.02	0.06	[41]
Non-GUD, treated	0.02	0.06	[41]

Abbreviations: GUD = genital ulcerative disease; non-GUD = non-ulcerative disease.

[a] Value multiplied by female-to-male transmissibility to determine male-to-female transmissibility.

[b] Primary HIV infection is assumed to last 0.2 years [10,35].

Table S3. Prevention interventions and their effects on behavior.[a]

	Reduction in non-use of condoms (%)			Reduction in non-treatment of STI (%)			Reduction in number of partners[b] (%)		Increase in age at first sex (years)
**Intervention**	**FSW**	**S**	**M**	**FSW**	**S**	**M**	**FSW**	**S**	
Mass media	..	17	17	..	..	..	..	..	..
VCT	50	34	16	..	..	..	..	..	..
Peer counseling – FSW	39	42	..	..	..	..	3	..	..
School-based programs	..	34	..	..	..	..	..	33	0.30
Workplace programs	39	34	1	..	..	..	..	23	..
Condom social marketing	21	11	5	..	..	..	..	..	0.12
Public sector condom distribution	57	10	5	11	..	..	35	..	..
STI treatment	54	14		47	22	..	50	..	..
pMTCT	50	34	16	..	..	..	..	..	..

Abbreviations: FSW = female sex worker; S = single men and women; M = married men and women; STI = sexually transmitted infection; VCT = voluntary counseling and testing programs for HIV/AIDS; pMTCT = prevention of mother-to-child transmission.

[a] See Bollinger et al. [19] for data sources and derivation of intervention effects. Effects are aggregated across all interventions to ascertain the average change in each behavior for each risk group, as described by Stover et al. [2]. Details on the calculations of condom use, STI treatment, partner numbers and age at first sex at a given level of intervention coverage are found in appendix 2 from ref. [2], available at http://image.thelancet.com/extras/02art6114webappendix2.pdf.

[b] Reductions in the probabilities that single men or married men visit sex workers are equal to the reduction in the number of partners for FSW.
